# Surgical Versus Non‐Surgical Treatment of Periodontitis: The Past, the Present, the Future

**DOI:** 10.1111/jre.70017

**Published:** 2025-07-15

**Authors:** Bruce L. Pihlstrom

**Affiliations:** ^1^ School of Dentistry University of Minnesota Minneapolis Minnesota USA

## 1 Introduction

Overall, the goal of periodontal therapy is to maintain the natural dentition in health, comfort, and function. For decades, dentists have considered, discussed, and debated the fundamental question of whether to use surgical or non‐surgical therapy to treat periodontitis [1, 2]. Historically, decisions for or against using various treatments were based on anecdotal evidence and clinical experience. Over the last 50 years or so, clinical research has provided objective data to support both methods of therapy. This led to the current management of periodontitis, which involves a combination of both non‐surgical and surgical interventions. One of the main questions facing clinicians today is how to monitor the stability of periodontitis following treatment so they can intervene to prevent or reverse further loss of periodontal support. While future treatment strategies cannot be predicted, advances in periodontal diagnosis, new technology, cost‐effectiveness, precision care, artificial intelligence, and new ways to control periodontal inflammation are likely to influence future methods of periodontal treatment.

## 2 The Past

### 2.1 Non‐Surgical Therapy

Non‐surgical therapy is the topic of another article in this series, but its overall purpose is to control periodontal inflammation. It includes local and systemic use of various medicaments, supra and subgingival instrumentation, scaling, root planing, and gingival curettage to remove epithelium and inflamed periodontal tissue adjacent to periodontal pockets. Gingival curettage was discontinued because it was found to offer no advantage over scaling and root planing alone [3] and minimally invasive non‐surgical therapy was introduced as a new approach for treating infra bony defects [4]. Many non‐surgical chemotherapeutic agents and procedures have been enthusiastically adopted and subsequently discarded when data proved them to be less effective than anticipated.

### 2.2 Surgical Therapy

Other than periodontal regeneration, which is discussed by another contributor in this series, the historical objective of periodontal surgery was to reduce periodontal pockets to a depth considered compatible with periodontal health. It included gingival resection and various flap procedures to debride root surfaces, remove granulation tissue, and reshape alveolar bone. Originally, flap surgery was used to remove necrotic and sequestered bone thought to be associated with periodontitis [5]. This concept was refuted in the 1930s when it was documented that bone associated with periodontitis was neither necrotic nor infected and did not need to be removed to achieve periodontal health [6, 7].

In the middle of the 20th century, the rationale for using flap surgery and osseous resection changed from removing “necrotic” bone to removing and reshaping it to create an underlying architecture consistent with shallow periodontal pockets [8]. The concept of using periodontal flaps to obtain access for root surface debridement adjacent to deep periodontal pockets was introduced as a modification of the Widman flap [9] and minimally invasive flap surgery was introduced for bone grafting [10].

## 3 Effectiveness of Non‐Surgical and Surgical Periodontal Therapy

In 1968 [11], investigators at the University of Michigan were the first to publish randomized clinical trials to test the effectiveness of various types of periodontal therapy. Known as the “Michigan Longitudinal Studies,” [12] the research generated intense criticism from academicians and clinicians who championed one type of treatment over others. The need to eliminate periodontal pockets deeper than 3 mm and the removal or reshaping of supporting bone were especially controversial [13]. Despite evidence from the Michigan studies to the contrary, strong opinions that pocket elimination surgery was essential for successful treatment of periodontitis persisted and many felt that non‐surgical treatment alone was ineffective and even unethical. Subsequent clinical trials and observational research in the United States and Europe confirmed and expanded results of the pioneering Michigan studies. Results from these studies were remarkably consistent and changed periodontal therapy as we know it today. Summarized in reviews [14, 15] and meta‐analysis [16, 17], the studies provided evidence that:

- Both surgical and non‐surgical periodontal therapy is effective in stabilizing clinical attachment level (CAL) [17]
- Non‐surgical subgingival debridement (scaling and root planing) results in greater CAL gain in moderately deep (4–6 mm) pockets and less CAL loss at shallow sites than access flap surgery [17].
- For moderate (4–6 mm) and deep (≥ 6 mm) pockets, both surgical and non‐surgical periodontal therapy is effective in reducing periodontal pockets and in stabilizing CAL [16, 17].
- Access flap surgery is more effective than non‐surgical therapy in reducing deep (≥ 6 mm) Periodontal pockets [17]
- There is no long‐term (> 3 years) difference in pocket depth reduction or stabilizing CAL between access and resective flap surgery [16].
- Resective osseous surgery to reshape periodontal tissues for the purpose of pocket elimination is not necessary to stabilize CAL [16].
- Pocket reduction to a specific pre‐specified depth is not necessary to stabilize CAL [16, 17].
- For intrabony defects, both minimally invasive surgical and non‐surgical treatment methods result in pocket reduction and CAL gain [4, 18, 19].
- Following periodontal treatment (irrespective of surgical or non‐surgical treatment) the overall percentage of teeth lost is very small (0%–2.4% from 60 to 156 months); most teeth lost are molars, and tooth loss is due to both periodontal and non‐periodontal causes [17].

It should be noted that these studies used practitioner‐measured outcomes of probing depth and CAL rather than the patient‐centered outcome of tooth retention. This was necessary because tooth loss from periodontitis occurs over many years, making it impractical to use as an outcome measure in shorter studies. However, CAL is generally accepted as a valid measure of periodontal support and, importantly, CAL loss ≥ 2 mm has been validated as an informative surrogate for tooth loss in a large 26‐year population study [20].

Additional studies have shown that:

- Subgingival debridement without flap access in deep pockets is technically demanding, making complete removal of calculus from root surfaces very difficult, if not impossible [21],
- Periodontal flap procedures facilitate access for removal of local irritants from root surfaces adjacent to deep periodontal pockets [21], and
- To be successful, periodontal therapy must control periodontal inflammation, which includes personal oral hygiene and regular professional supportive care [22].

## 4 The Present

In the past, surgical and non‐surgical treatment for periodontitis were often viewed as distinct and separate treatment strategies. Today there is more emphasis on integrating non‐surgical and surgical treatment into a continuum of therapy. Depending on specific diagnoses, systemic health, risk factors, and other considerations, both are frequently used and have been endorsed by the American Academy of Periodontology [23] and the European Federation of Periodontology [24].

### 4.1 Non‐Surgical Therapy

Non‐surgical periodontal treatment has been described as the “Gold Standard” of periodontal therapy [25]. It is widely recognized as one of the first steps in treatment for periodontitis, and its outcome is the basis for making decisions for additional therapy. In a major shift away from past surgical treatment of moderate periodontitis, patients having 4–6 mm pockets are now routinely treated non‐surgically by both dentists and dental hygienists.

### 4.2 Surgical Therapy

Surgical periodontal therapy is widely used today for the treatment of advanced periodontitis. It involves flap access, resective, and regenerative surgery. Flap access surgery provides access to root surfaces adjacent to deep periodontal pockets for the removal of dental calculus, while resective surgery has the primary purpose of eliminating pockets or reducing probing depth. However, resective pocket elimination surgery results in increased gingival recession and can lead to tooth sensitivity, root caries, and compromised esthetics. It may also result in more tooth loss because teeth having deep pockets, which cannot be eliminated, may be extracted even though they could be retained in comfort and function. Overall, the results of pocket elimination surgery may be patient dissatisfaction and less compliance with critical follow‐up supportive care, along with higher patient costs for dental implants or prostheses required to replace extracted teeth. These factors are rarely considered in treatment recommendations or professional guidelines.

As noted earlier, evidence from numerous clinical trials consistently indicates that neither resective nor flap access surgery is more effective than non‐surgical treatment for stabilizing CAL and tooth retention. However, there is some evidence indicating that access flap surgery may reduce the need for retreatment due to progressive loss of clinical attachment at specific sites [26]. While current European professional guidelines suggest the use of flap access or resective surgery for treating residual pockets greater than 6 mm [24], there is an emerging trend to use flap access surgery, minimally invasive methods, and periodontal regeneration rather than surgical pocket elimination.

### 4.3 Making Decisions for Periodontal Surgery

In the past, decisions for or against periodontal surgery were often made when periodontitis was first diagnosed. Today, most practitioners make this decision following initial non‐surgical treatment using residual probing depth and bleeding on probing (BOP). The European Federation of Periodontology S3 level practice guideline recommends that the third step of periodontal therapy be implemented if the endpoints of therapy (no periodontal pockets > 4 mm with BOP or deep pockets ≥ 6 mm) have not been achieved after an adequate healing period following stage II therapy [24]. The guidelines suggest repeating subgingival instrumentation for residual 4–5 mm pockets and periodontal surgery for deep residual pockets (≥ 6 mm) [24]. However, the use of probing depth and BOP as reliable indicators of progressive disease or lack of periodontal stability may be questioned. While there is limited evidence that patients having advanced periodontitis with multiple residual probing depths > 6 mm are at greater risk of additional attachment loss than patients with fewer such sites [27], other evidence indicates that probing depth is not a reliable predictor of CAL change [28, 29]. Moreover, while there is good evidence that lack of BOP predicts CAL stability [30], the presence of BOP has limited use as a prognostic indicator of stability [31].

It is also unclear if probing depth and BOP are useful primary treatment endpoints over time. For example, a recent study reported that among untreated patients who were followed for 12 months, there was little difference in BOP between those having progressive CAL loss and those with stable CAL (45.3% ± 23.0% vs. 42.8% ± 24.3%, respectively) [32]. Six months following non‐surgical periodontal therapy, the percentage of sites having BOP decreased for both groups of patients but again, there was little difference in percentage BOP between those having progressive periodontitis and those having stable periodontitis (29.5% ± 17.5% vs. 30.9% ± 21.0%, respectively) [32]. If the goal of periodontal therapy is to achieve periodontal stability, these data question the commonly held concept that successful treatment for periodontitis requires ≤ 10% BOP [24, 33]. On the other hand, CAL loss precedes radiographic evidence of bone loss during periods of disease activity and CAL measurement has high predictive discrimination for alveolar bone loss [34]. Moreover, as noted earlier, loss of attachment ≥ 2 mm has been shown to be a valid surrogate measure for tooth loss over time [20]. These findings challenge the common belief that primary treatment endpoints based on probing depth and/or BOP should be met to control “active” periodontitis by either surgical or non‐surgical treatment.

The bottom line is that CAL stability should be the primary endpoint for treatment of periodontitis. Probing depth and BOP should be used as secondary endpoints. Following treatment of periodontitis, CAL should be carefully monitored and used for making decisions about additional therapy that may be needed during follow‐up supportive care. Measuring CAL is technically difficult and time‐consuming, but unless CAL is measured and monitored over time, clinicians risk failing to identify teeth that gain or lose clinical attachment [28]. By not measuring CAL, they also risk missing the opportunity to intervene by either surgical or non‐surgical means to arrest or reverse loss of clinical attachment.

## 5 The Future

It is impossible to predict the future of any discipline, but current trends can provide some insight into what may transpire in coming years. Regardless of future treatment methods, controlling periodontal inflammation and the oral biofilm will remain essential for successful surgical and non‐surgical periodontal therapy. New developments in helping patients improve oral hygiene and comply with supportive care and new ways to change harmful behaviors such as substance and tobacco use could have profound effects on periodontal treatment methods. Rather than using a periodontal probe and laborious methods of physical clinical measurement (i.e., CAL, probing depth, BOP), future clinicians will likely use improved diagnostic methods and biomarkers that will allow precise identification of patients or sites within patients at risk for progressive periodontitis in real time. There is also a significant potential for artificial intelligence to have a central role in future periodontal diagnosis, prognosis, and treatment [35, 36, 37]. Today, generic periodontal treatment plans are used for most patients, but there is recent evidence that not all individuals respond equally to periodontal therapy because of varying environmental, microbial, immunological, and systemic health conditions [38]. Given rapid advances in artificial intelligence, one can easily imagine a future when artificial intelligence uses specific patient and periodontal information to make data‐driven diagnoses and recommend precision treatment that is personalized for individual patients. Minimally invasive non‐surgical and surgical treatments, microsurgery, and periodontal regenerative procedures are showing promise [4, 19, 39, 40]. As new technologies become available, it is likely that future periodontal therapy will be less invasive and more targeted at specific periodontal sites that are progressive or more likely to respond to treatment. Coupled with new developments in periodontal regeneration, this could result in increased cost‐effectiveness, better patient acceptance and adherence to periodontal therapy, fewer treatment complications, less patient discomfort, and better treatment outcomes. Overall, the future is likely to bring advances in periodontal therapy that will likely render the distinction between surgical and non‐surgical therapy irrelevant and mainly of historical interest.

## Data Availability

There is no original data presented in this manuscript. All data presented has been published in the public domain and has been cited in the bibliography.
